# Hippocampal dysfunction after autoimmune encephalitis depending on the antibody type

**DOI:** 10.1007/s00415-024-12742-1

**Published:** 2025-02-01

**Authors:** Martin Hänsel, Heinz Reichmann, Antje Haehner, Henning Schmitz-Peiffer, Hauke Schneider

**Affiliations:** 1 Department of Neurology, University of Dresden, Fetscherstraße 74, 01307 Dresden, Germany; 2 Department of Internal Medicine, GZO-Zurich Regional Health Center, Wetzikon, Switzerland; 3 Department of Otorhinolaryngology, Medical Faculty Carl-Gustav Carus, Smell and Taste Clinic, Technical University of Dresden, Dresden, Germany; 4 Department of Neurology, Augsburg University Hospital, Augsburg, Germany

**Keywords:** Autoimmune encephalitis, Neurocognition, Hippocampus, Incidental learning, RVDLT, VLMT, MoCA, NMDA, GAD, LGI1, Caspr2

## Abstract

**Background:**

Comprehensive neurocognitive function analyses of autoimmune encephalitis (AE) patients, especially long-term ones, are rare. This study aims to measure cognitive function in patients diagnosed with AE.

**Methods:**

This case–control study included AE patients (*n* = 11) with antibodies against NMDA receptor (NMDAR) (*n* = 4), VGKC (*n* = 3), GAD (3), and one antibody-negative patient. The control group contained 12 pneumococcal meningo-encephalitis patients (PC). Subgroup analyses compared AE patients with and without NMDAR antibodies. Neurocognitive tests were performed to evaluate verbal and visual memory, face recognition, attentional capacity, incidental learning capacity, and overall cognitive function (Montreal cognitive assessment, MoCA). Limbic structural involvement was assessed through magnetic resonance imaging (MRI). Statistical analyses investigated correlations between antibody status, results of neurocognitive tests, and MRI findings.

**Results:**

Follow-up (AE vs. PC) was 33 (11–95) vs. 96 (26–132) months after diagnosis. Neurocognitive functions were normal in both AE and PC groups in all tests except face recognition, which was pathological in both groups. The overall/recognition/long-delay visual memory (*p* = 0.009/0.008/0.005) and incidental learning (*p* = 0.017) scores were significantly higher in NMDAR patients compared to non-NMDAR patients. Non-NMDAR patients with right-sided limbic MRI pathologies had significantly lower overall/recognition/long-delay visual memory (*p* = 0.006/0.044/0.024) and incidental learning (*p* = 0.009) scores compared to NMDAR patients.

**Conclusions:**

We observed mainly normal neurocognitive functions after autoimmune and bacterial encephalitis. However, compared to NMDAR patients, patients with non-NMDAR autoimmune encephalitis showed a significant and material-specific association between a right-sided hippocampal lesion and limitations in figural-mnestic and incidental learning capacities. Neurocognitive functions in AE patients should be further evaluated prospectively and in more detail.

**Supplementary Information:**

The online version contains supplementary material available at 10.1007/s00415-024-12742-1.

## Introduction

Medial temporal lobe abnormalities, especially involvement of hippocampal structures, are a well-described imaging finding in patients with autoimmune encephalitis (AE) contributing to the syndrome of limbic encephalitis [1, 2]. Therefore, neurocognitive deficits are well-known symptoms in AE patients [1, 3–11]. Depending on the antibody type and the time of cognitive testing after symptom onset, different cognitive impairments were described for AE patients. In one study, 64 of 75 (85%) patients with anti-N-methyl-D-aspartate receptor (NMDAR) encephalitis had frontal lobe impairments such as problems with attention and planning, impulsivity, and behavioural disinhibition after discharge [3]. Chen et al. found anterograde amnesia, emotional lability, and attentional difficulties among 4 of 16 patients with NMDARs one year after diagnosis [12]. A very extensive neuropsychological assessment showed deficits in attention (4/9), working memory (4/9), episodic memory (2/9), and executive functioning (5/9) in an NMDAR group of 9 patients at a median of 43 months after disease onset [4]. The most comprehensive longitudinal neuropsychological assessment of 43 NMDAR patients showed cognitive deficits in all patients 2 years after onset, with improvement in all cognitive domains 4.9 years after onset, but with persistent deficits in memory and executive functions [10]. AE patients with voltage-gated potassium channel (VGKC) antibodies also showed significant impairment in memory, processing speed, and executive function early during disease course and prior to immunotherapy; however, at follow-up, processing speed and executive function returned to normal, while cognitive impairment was limited to memory [13]. Neurocognitive outcomes in AE patients with autoantibodies to glutamic acid decarboxylase (GAD) were studied by Frisch et al. In contrast to VGKC patients, GAD patients showed less impaired learning and memory, but no improvement of these cognitive functions with immunotherapy [14]. Data on incidental learning, which is a mixture of attention, working memory, episodic memory, and executive function, are not available for AE patients to our knowledge.

The aim our study was to measure cognitive functions in patients after autoimmune encephalitides associated with different AE antibodies and in a comparison group of patients with bacterial meningitis. Furthermore, we aimed to compare cognitive functions and imaging findings in AE subgroups (NMDAR vs. non-NMDAR patients).

## Methods

### Patients

We designed a single-center, case–control study to compare AE patients with sex- and age-matched patients with pneumococcal meningo-encephalitis (PC), treated at the Universital Hospital Dresden between January 2002 and March 2016. Patients were selected by retrospective review of electronic databases and chart review. The inclusion and exclusion criteria for AE and PC patients are described in detail in the Supplementary Material (Supplementary Figure S1). Established diagnostic criteria for possible AE were used as proposed by Graus et al., based on positive antibody findings in serum and/or cerebrospinal fluid as well as clinical symptoms [15]. The AE group includes 4 patients with antibodies to NMDAR, 3 with antibodies to VGKC, 3 with antibodies to GAD and 1 antibody-negative patient. Antibodies to NR1/NR2B heteromers of the NMDAR were detected through indirect immunofluorescence on NR1/NR2B transfected human embryonic kidney cells for AE patients diagnosed in 2010 and 2011 [16, 17]. For all other AE patients, the antibodies were detected by indirect immunofluorescence on commercially available mouse brain tissue and cell-based assays (Euroimmun, Lübeck, Germany) [18]. Subgroup analyses between the NMDAR group and the non-NMDAR group (VGKC, GAD, antibody-negative) were performed. At the time of the initial treatment phase of study patients, routine tests were available for anti-VGKC, but not for anti-LGI1 and anti-Caspr2 for further differentiation.

All patients gave written informed consent. The study was approved by the ethics committee of the University of Dresden. Clinical variables and imaging data were analysed, followed by clinical follow-up between August and December 2016 for the comprehensive neurocognitive tests.

## Clinical and functional variables of AE and PC patients in acute and post-acute phase

The following parameters were included: (1) demographics (sex, age), (2) clinical symptoms, (3) intensive care unit (ICU) treatment, (4) modified Rankin Scale (mRS) scale at disease onset and follow-up, (5) disease-specific treatment, (6) detection of malignancies, (7) MRI results regarding side of limbic MRI pathologies (e.g., temporomesial, temporobasal, hippocampus, and corpus amygdaloideum), (8) length of hospital stay, (9) relapses, (10) immunotherapy and antiepileptic therapy at follow-up, (11) anamnestic neurocognitive deficits at follow-up, and (12) learning capacity at follow-up.

Available cerebral MR images (FLAIR- and T2 sequences), performed for routine clinical care, were evaluated by a senior neuroradiologist. Three different MRI scanners were used (Siemens Magnetom Verio, 3.0 Tesla; Siemens Magnetom Vision, 1.5 Tesla; GE Signa HDxt, 3.0 Tesla), depending on the availability of the scanner in the clinical routine.

## Neurocognitive assessment at follow-up

A cross-sectional study of recruited AE and PC patients was performed and included a neurological examination and neuropsychological testing. To characterise neurocognitive abilities, the following parameters were assessed: (1) Current anamnestic cognitive problems, (2) handedness, (3) aphasia score, and (4) Hospital Anxiety and Depression Scale (HADS). The aphasia score was used to rule out aphasia, potentially interfering with neurocognitive testing (Supplementary Figure S2). The HADS was used to exclude a possible depression or anxiety as an indication of pseudo-dementia syndrome [19]. To assess verbal memory as a left hemispheric and left temporal function, such as verbal short-term and working memory, we used the Verbal Learning Memory Test (VLMT) [20]. Visual capacity was assessed using the Rey Visual Design Learning Test (RVDLT) and Alsterdorfer Faces Test (AFT) to assess right hemisphere functions including right temporal capacity for short and long memory [21–23]. Attentional capacity was measured using the Digit Symbol Substitution Test (DSST) as a correlate of the frontal lobe (Supplementary Material). To measure incidental learning capacity, we extended the DSST with a 2-min delay incidental learning query. The Montreal Cognitive Assessment Test (MoCA) was used to provide an overall impression of the patients' neurocognitive function and also to screen for mild cognitive impairment. [24]

## Statistical analyses

The unpaired t test was used for metric data with a 95% confidence interval and reported as significant when p < 0.05. Nominal data were analysed using Fisher's exact test. The Mann–Whitney U test was used to analyse significant relations between pathological MRI findings and neurocognitive outcomes, the unpaired t test for metric data. The effect sizes for neurocognitive function were calculated using Cohen's d. We used the Shapiro–Wilk test due to the small sample size. The distribution results are presented in the Supplementary Material. Statistical analyses were performed using SPSS (V.23.0, IBM, Armonk, New York, USA).

## Results

### Clinical and functional variables in AE and PC patients in acute and post-acute phase

The median age (31 years, range 17–74) and sex distribution (8 women) of the 11 AE patients were not significantly different from the 12 PC patients (median age 41 years, range 26–52; 5 women). Of all AE patients, 8/11 had seizures, 4/11 patients extrapyramidal motoric symptoms, and 2 had pyramidal symptoms (Table 1). Furthermore, 9/11 AE patients suffered from cognitive symptoms, 5/11 from psychiatric symptoms, and 6/11 patients had autonomic dysregulations. A tumour was found in one AE patient (right amygdala and uncus gyri hippocampi) at onset and in further 3/11 patients (incidentaloma) at follow-up. ICU treatment was required in 6/11 AE patients with a median mRS of 3 points (range 2–5). Initially, 9/11 patients were treated with intravenous methylprednisolone, 4/9 patients in combination with intravenous immunoglobulins (IVIG), and 2/9 in combination with immunoadsorption. From 4 of these 9 patients with first-line therapy, a second-line therapy with Rituximab was needed. The remaining 2/9 patients without methylprednisolone therapy were treated with antiepileptic drugs (detection of GAD antibodies was years later). The mean hospitalisation duration was 27.1 ± 15.8 days in the AE group and significant longer compared to the PC control group (15.7 ± 4.1 days, p = 0.039).

**Table 1:** Patient characteristics in AE and PC groups

	AE group	PC group	*p* value
Patients, n	11	12	–
Ratio female: male	8:3	5:7	*p* = 0.214
Age, at diagnosis (years), median [range]	31 [17–74]	41 [26–52]	*p* = 0.984
Antibodies against, n			
Synaptic receptors: NMDAR	4	–	–
Ion channels: VGKC (LGI1, Caspr2)	3	–	–
Intracellular antigen: GAD	3	–	–
Antibody negative	1	–	–
Intensive care unit stay, n	6	12	*p* = 0.014
mRS score, median [range]			
Acute (maximum mRS score)	3 [2–5]	5 [2–5]	*p* = 0.014
Follow-up	1 [0–1]	1 [0–4]	*p* = 0.236
Acute therapy, n			
Intravenous corticosteroids	9	12	0.217
Intravenous immunoglobulins	4	0	–
Immunoadsorption	2	0	–
Second line therapy, n			
Rituximab	4	0	–
Limbic MRI pathologies, n	6	0	*p* = 0.018
Hospitalisation time (d), mean, [range]	27.1 [6–48]	15.7 [10–25]	*p* = 0.039
Time from diagnosis to follow-up (mo), median, [range]	33 [11–95]	96 [26–132]	*p* = 0.008
Relapse, n	2	0	*p* = 0.217
Therapy at follow-up, n			
Immunotherapy	5	0	*p* = 0.014
Antiepileptic drugs	5	0	*p* = 0.014
Anamnestic, neurocognitive symptoms at follow-up, n	6	7	*p* = 1.000
Fully employed, if not retired at follow-up, n	9	11	*p* = 0.590

*AE Autoimmune encephalitis; Caspr2 Contactin-associated protein-like 2; d Days; GAD Glutamate acid decarboxylase; HADS-D Hospital anxiety and depression scale; LGI1 Leucine-rich glioma inactivated 1; mo Months; MoCA Montreal cognitive assessment; mRS Modified Ranking Scale; n Number; NMDAR N-methyl-D-aspartate Receptor; PC Pneumococcal meningo-encephalitis; VGKC Voltage-gated potassium channels, yrs Years*

Initially, pathological MRI findings in limbic structures were found in 6/11 AE patients, which persisted in all available follow-up MRIs (median time of 4 months, range 1–71). Relapse was noted in 2 GAD patients. At follow-up, 4 AE patients were treated with oral prednisolone, one patient in combination with azathioprine. One AE patient was treated (repeatedly) with IVIG. Antiepileptic drug therapy was ongoing in 5 patients. Of all AE patients at the follow-up, 6/11 were fully employed, 4 patients in retirement and one got disability pension.

### Neurocognitive function at follow-up in AE and PC patients

Follow-up after diagnosis was 33 (11–95) months in the AE group and 96 (26–132) months in the PC group. At follow-up, 6/11 patients with AE and 7/12 patients with PC reported cognitive problems like short- and long-term memory problems, impaired face recognition, amnesia, fears of failure, and impairment in multitasking skills (p = 1.000) (Table 2). All patients were right-handed except one NMDAR patient. The aphasia score, the HADS-D, and the HADS-A did not differ significantly (Supplementary Material). The overall verbal memory performance (VLMT) was in both groups (AE vs. PC) similar with a median of 52 (range 34–62) points vs. 51 (range 36–68) points (p = 0.874). The verbal recognition performance did not differ significantly between the AE (11 points, range 5–15) and PC group (13.5 points, range 10–15) (p = 0.088). The median overall visual performance (RVDLT) was not different in AEs (30 points, range 12–56) compared to PC patients (44.5 points, range 20–62), (p = 0.193). Furthermore, the visual recognition performance (p = 0.378) was not different in the AE group (11 points, range 6–15) compared to the PC group (13 points, range 8–15). The median percentage visual memory performance, using the AFT, was comparable in the AE group (55%, range 30–95) and in the PC group (65%, range 15–100) (p = 0.830). The median attentional capacity (DSST) was not different in the AE group (58 points, range 20–77) and in the PC group (46.5 points, range 28–64) (p = 0.421). Furthermore, incidental learning was comparable in both groups (AE: median score 5, range 0–9; PC: median score7.5, range 1–9) (p = 0.220). Cognitive function according to MoCA was similar in both groups, 27 (range 17–29) points in the AE group vs. 27.5 (range 22–30) points in the PC group (p = 0.281).

**Table 2:** Neurocognitive function at follow-up in AE and PC groups

	AE group	PC group	p value	Effect size
VLMT overall, mean (SD)	49.6 (8.6)	50.3 (9.7)	*p* = 0.874	*d* = −0.076
VLMT recognition, mean (SD)	11.1 (3.3)	13.0 (1.7)	*p* = 0.088	*d* = −0.734
RVDLT overall, mean (SD)	33.6 (15.2)	41.3 (12.6)	*p* = 0.193	*d* = −0.554
RVDLT recognition, mean (SD)	10.9 (3.3)	12.0 (2.5)	*p* = 0.378	*d* = −0.378
AFT, mean (SD)	59.1 (20.8)	57.1 (23.3)	*p* = 0.830	*d* = 0.090
DSST, mean (SD)	52.0 (18.9)	46.6 (12.3)	*p* = 0.421	*d* = 0.342
DSST incidental, mean (SD)	5.1 (3.3)	6.7 (2.7)	*p* = 0.220	*d* = −0.533
MoCA, mean (SD)	25.8 (3.3)	27.2 (2.6)	*p* = 0.281	*d* = −0.474

*AE Autoimmune encephalitis; AFT Alsterdorfer faces test; DSST Digit symbol substitution test; MoCA Montreal cognitive assessment; PC Pneumococcal meningo-encephalitis; RVDLT Rey visual design learning test; SD Standard deviation; VLMT Verbal learning memory test*

### MRI findings in AE and PC patients

**Fig. 1:**
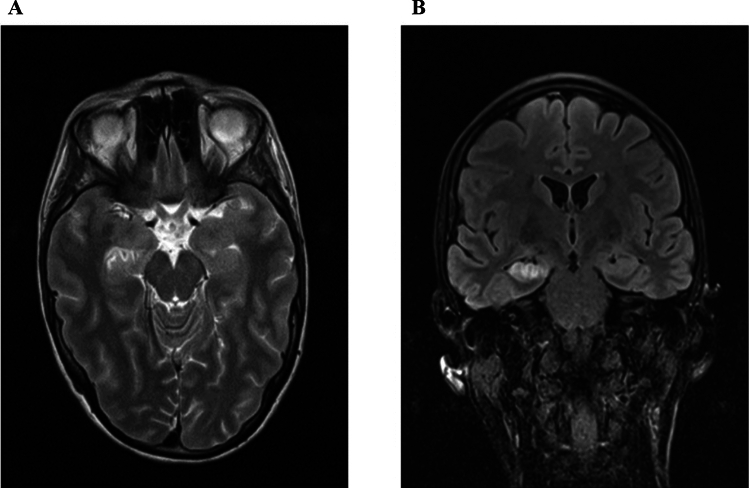
Limbic MRI pathologies in a patient with GAD antibodies, Representative MRI findings in a patient with right-hippocampal necrosis and temporomesial space-occupying effects in the right amygdala complex, hippocampus, and nucleus accumbens. **A** T2-weighted sequence, axial; **B** FLAIR-sequence, coronary (source: University Hospital Dresden, Department of Neuroradiology)

In 6/11 AE patients, limbic MRI pathologies were present at the time of initial hospital MRI and at all follow-up MRIs [median time 4 (range 1–71) months] (Fig. 1). The remaining 5/11 AE patients and all PC patients had no limbic MRI pathologies both in the early phase and during all follow-ups (p = 0.018). The limbic MRI pathologies were on the right hemisphere in 5/6 patients and on the left hemisphere in one patient. Patients with limbic MRI pathologies (n = 6/19) had a significantly worse visual figural function in the overall RVDLT (25.5 ± 13.6, p = 0.018) compared to the patients without limbic MRI pathologies (41.5 ± 11.8, n = 13/19). Furthermore, the group of patients with limbic MRI pathologies, compared to patients without MRI pathologies, had significantly lower incidental learning results in the DSST (p = 0.005). Patients with limbic MRI pathologies had a mean incidental DSST score of 3.2 (± 3.1), the group without a score of 7.0 (± 2.0).

### Clinical and functional variables in NMDAR and non-NMDAR patients in acute and post-acute phase

Patients of the NMDAR group (4/4 women, median age 19 years, range 17–29) were significantly younger at disease onset than patients of the non-NMDAR group (4/7 women, median age 58 years, range 23–74) (p = 0.006). The median length of hospital stay was significantly different between the two groups (NMDAR vs. non-NMDAR: 42 days vs. 15 days) (p = 0.023) (Table 3). The results of antibody testing in cerebrospinal fluid and serum in NMDAR and non-NMDAR groups in acute and post-acute phase are reported in the Supplementary material (Supplementary Table 1).

**Table 3:** Patient characteristics in NMDAR and non-NMDAR groups

	NMDAR patients	non-NMDAR patients	p value
Patients, n	4	7	–
Ratio female: male	4: 0	4: 3	*p* = 0.236
Age, at diagnosis (years), median [range]	19 [17–29]	58 [23–74]	*p* = 0.006
Clinical symptoms, n			
Seizure	3	5	*p* = 1.000
Extra pyramidal motoric symptoms	3	1	*p* = 0.088
Pyramidal symptoms	0	2	*p* = 0.491
Cognitive symptoms	4	5	*p* = 0.491
Psychiatric symptoms	3	2	*p* = 0.242
Autonomic dysregulation	2	4	*p* = 1.000
Tumour, n	1	3	*p* = 1.000
Intensive care unit stay, n	4	2	*p* = 0.061
mRS score, median [range]			
Acute (maximum mRS score)	3 [3–5]	2 [2–4]	*p* = 0.166
Follow-up	0 [0–1]	1 [0–1]	*p* = 0.166
Acute therapy, n			
Intravenous corticosteroids	4	5	*p* = 0.491
Intravenous immunoglobulins	3	1	*p* = 0.088
Immunoadsorption	0	2	*p* = 0.491
Second line therapy, n			
Rituximab	3	1	*p* = 0.088
Limbic MRI pathologies, n			
Right side: left side	0	5: 1	*p* = 0.015
Hospitalisation time (d), mean, [range]	40.5 [33–45]	19.4 [6–48]	*p* = 0.023
Time from diagnosis to follow-up (mo), median, [range]	83 [20–95]	28 [11–85]	*p* = 0.121
Relapse, n	0	2	*p* = 0.491
Therapy at follow-up, n			
Immunotherapy	0	5	*p* = 0.061
Antiepileptic drugs	0	5	*p* = 0.061
Anamnestic, neurocognitive symptoms at follow-up, n	1	5	*p* = 0.242
Fully employed, if not retired at follow-up, n	3	6	*p* = 1.000

*Caspr2 Contactin-associated protein-like 2; d days; GAD Glutamate acid decarboxylase; HADS-D Hospital anxiety and depression scale; LGI1 Leucine-rich glioma inactivated 1; mo Months; MoCA MONTREAL cognitive assessment; mRS Modified ranking scale; n Number; NMDAR N-methyl-D-aspartate Receptor; non-NMDAR Without N-methyl-D-aspartate Receptor; PC Pneumococcal meningo-encephalitis; VGKC Voltage-gated potassium channels; yrs Years*

### Neurocognitive function at follow-up in NMDAR and non-NMDAR patients

**Fig. 2:**
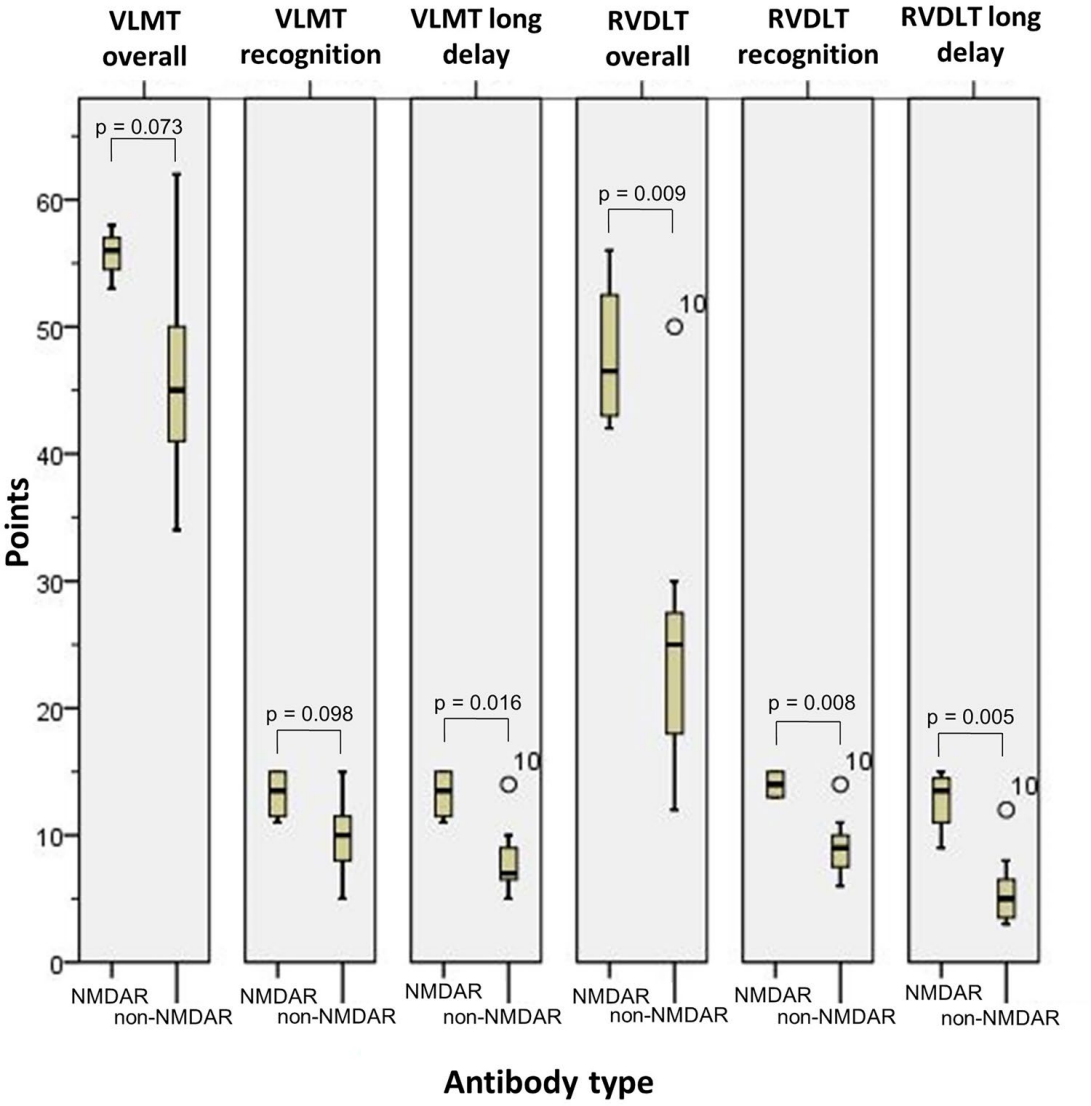
VLMT overall, recognition and long delay, RVDLT overall, recognition, and long delay in NMDAR and non-NMDAR patients *NMDAR* N-methyl-D-aspartate Receptor; *non-NMDAR* without N-methyl-D-aspartate Receptor; *RVDLT* Rey visual design learning test; *VLMT* Verbal learning memory test

**Fig. 3:**
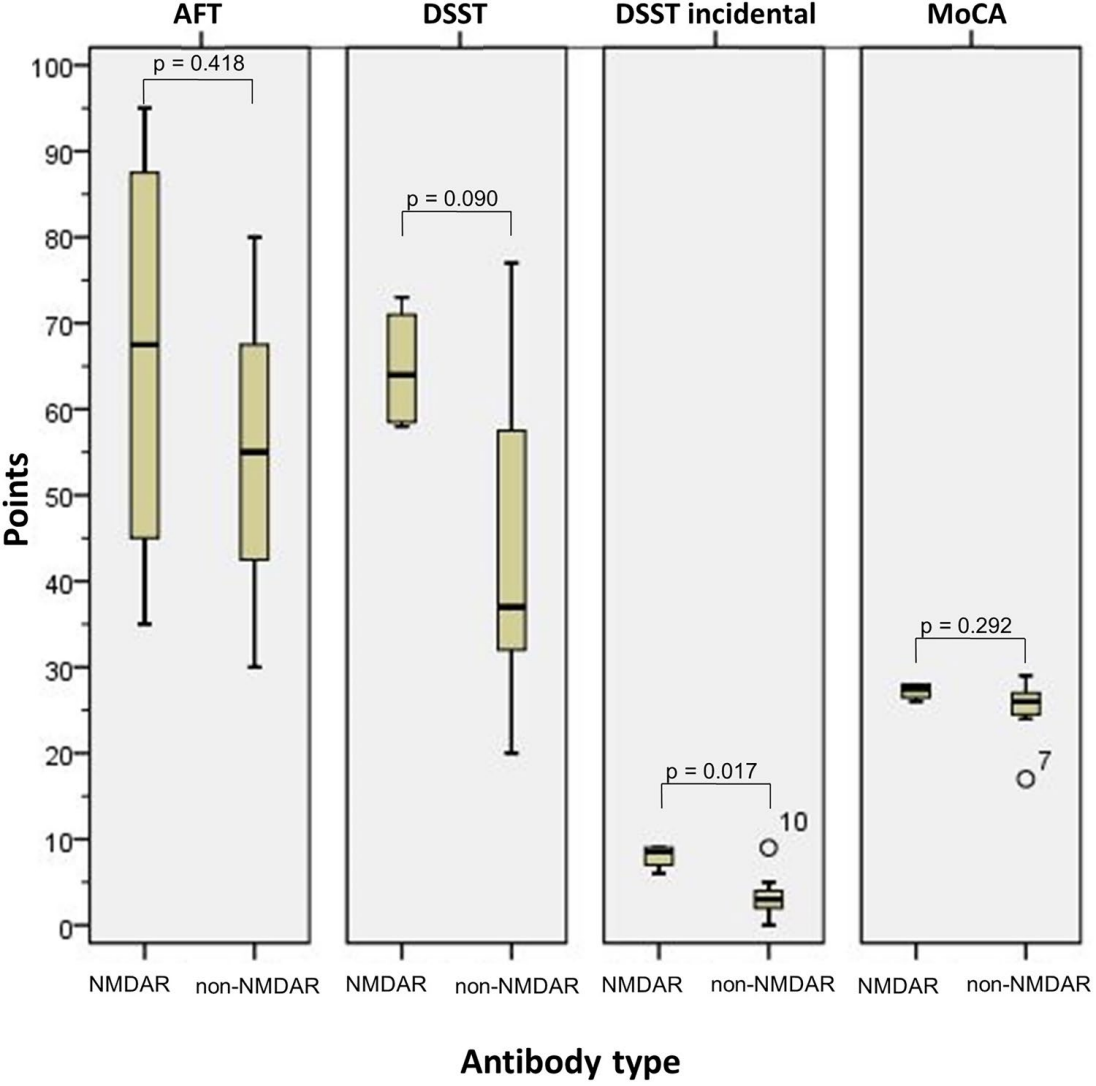
AFT, DSST overall and incidental and MoCA in NMDAR and non-NMDAR patients *AFT* Alsterdorfer faces test; *DSST*, Digit symbol substitution test; MoCA Montreal cognitive assessment; *NMDAR* N-methyl-D-aspartate Receptor; *non-NMDAR* without N-methyl-D-aspartate Receptor

Follow-up after diagnosis was 83 (20–95) months in the NMDAR group and 28 (11–85) months in the non-NMDAR group. At follow-up, 1/4 NMDAR patient reported short-term memory problems, whereas 5/7 non-NMDAR patients reported anamnestic cognitive problems in short- and long-term memory, in multitasking skills, fears of failure, and face recognition (p = 0.242). The aphasia score and levels of HADS-A and HADS-D were not significantly different (Supplementary Material). The NMDAR group had a not significant (p = 0.073) different overall verbal memory performance (VLMT) (median 56 points, range 53–58) compared to the non-NMDAR group (median 45 points, range 34–62). (Table 4). Furthermore, the verbal memory recognition test showed no significant difference (p = 0.098) between the NMDAR group (median 13.5 points, range 11–15) and the non-NMDAR group (median 10.0 points, range 5–15). NMDAR patients showed a statistically significant higher median (13.5 points, range 11–15) in the long recall of the VLMT (p = 0.016) compared to the non-NMDAR patients (median 7 points, range 5–14) (Fig. 2). In the overall visual performance (RVDLT), the NMDAR group (median 46.5 points, range 42–56) performed better than the non-NMDAR group (median 25 points, range 12–50) (p = 0.009). Moreover, the NMDAR patients showed a significant difference (p = 0.008) in the visual recognition performance test (median 14 points, range 13–15) compared to the non-NMDAR patients (median 9 points, range 6–14). Also, NMDAR patients had a significantly higher median (13.5, range 9–15) in the long-recall RVDLT (p = 0.005) compared to the non-NMDAR patients (median 5 points, range 3–12). The visual performance, measured by the AFT, was not significantly different between the NMDAR patients with a median of 67.5 points (range 35–95) compared to the non-NMDAR patients who scored a median of 55 points (range 30–80) (p = 0.418) (Fig. 3). Thereof, only 2/4 of the NMDAR patients and 1/7 of non-NMDAR patients achieved a normal AFT result. There were no significant differences (p = 0.090) in the DSST scores between the NMDAR group (median 64 points, range 58–73) and the non-NMDAR group (median 37 points, range 20–77). The incidental learning was significantly better in the NMDAR group (median 8.5 points, range 6–9) than in the non-NMDAR group (median 3 points, range 0–9) (p = 0.017). The MoCA results were similar in both groups with 27.5 (range 26–28) points in the NMDAR group vs. 26 (range 17–29) points in the non-NMDAR group) (p = 0.292).

**Table 4:** Neurocognitive function in NMDAR and non-NMDAR groups

	NMDAR group	non-NMDAR group	*p* value	Effect size
VLMT overall, mean (SD)	55.8 (2.1)	46.1 (9.1)	*p* = 0.073	*d* = 1.289
VLMT recognition, mean (SD)	13.3 (2.1)	9.9 (3.3)	*p* = 0.098	*d* = 1.151
VLMT long delay, mean (SD)	13.3 (2.1)	8.1 (3.0)	*p* = 0.016	*d* = 1.903
RVDLT overall, mean (SD)	47.8 (6.2)	25.4 (12.4)	*p* = 0.009	*d* = 2.086
RVDLT recognition, mean (SD)	14.0 (1.2)	9.1 (2.7)	*p* = 0.008	*d* = 2.120
RVDLT long delay, mean (SD)	12.8 (2.6)	5.7 (3.3)	*p* = 0.005	*d* = 2.302
AFT, mean (SD)	66.3 (26.6)	55.0 (17.8)	*p* = 0.418	*d* = 0.534
DSST, mean (SD)	64.8 (7.4)	44.7 (20.0)	*p* = 0.090	*d* = 1.191
DSST incidental, mean (SD)	8.0 (1.4)	3.4 (2.9)	*p* = 0.017	*d* = 1.839
MoCA, mean (SD)	27.3 (1.9)	25.0 (3.9)	*p* = 0.292	*d* = 0.683

*AFT Alsterdorfer Faces Test; DSST Digit symbol substitution test; MoCA Montreal cognitive assessment; NMDAR N-methyl-D-aspartate Receptor; non-NMDAR Without N-methyl-D-aspartate Receptor; RVDLT Rey visual design learning test; SD Standard deviation; VLMT Verbal learning memory test*

### MRI findings in NMDAR and non-NMDAR patients

In contrast to 6 out of 7 patients in the non-NMDAR group, none of the 4 NMDAR patients exhibited limbic MRI pathologies at the onset of the disease or in any follow-up MRIs [mean follow-up time of 13.3 (± 21.7) months; p = 0.015]. Patients with a limbic MRI pathology on the right side (n = 5) compared to those without limbic MRI pathologies (n = 5) had significant worse results in the overall RVDLT [mean 20.6 (± 7.1) vs. 43.2 (± 11.5), p = 0.006], recognition RVDLT [mean 8.6 (± 1.8) vs. 12.6 (± 3.3), p = 0.044], long-delay RVDLT [mean 4.8 (± 2.0) vs. 11.0 (± 4.5), p = 0.024], long-delay VLMT [mean 7.2 (± 1.9) vs. 12.0 (± 3.3), p = 0.023], and incidental DSST [mean 2.0 (± 1.2) vs. 7.4 (± 1.8), p = 0.001). The median duration between the last MRI and neurocognitive test for the five patients with limbic pathologies on the right hemisphere was 25 (range 11–69) months.

## Discussion

Neuropsychiatric symptoms and prolonged neurocognitive deficits in AE patients and its relationship especially with impaired limbic functions were described in several studies [1, 3–11]. In addition to these studies, we were able to show a predominant right-hippocampal dysfunction and impaired incidental learning after autoimmune encephalitis, depending on the AE antibody type. Overall cognitive performance at long-term follow-up was normal in the AE group, except for the visual capacity assessed using the Alsterdorfer Faces Test. However, in subgroup analyses, the NMDAR group performed numerically better than the non-NMDAR group on almost all neurocognitive tests and significantly better on right-hippocampal functions (RVDLT) and incidental learning (DSST). Additional analyses showed that AE patients with right-sided limbic MRI pathologies had significantly worse results on the overall/recognition/long-delay RVDLT and worse incidental learning skills (DSST) compared to those without limbic MRI pathologies. To our knowledge, these findings were not previously reported in AE patients.

Several aspects of our findings need to be discussed. First, NMDAR patients may experience greater neurocognitive improvement following AE in comparison to non-NMDAR patients, potentially attributable to differing pathophysiological pathways in these AE subgroups. Due to antibody-mediated capping and internalisation of NMDARs from the cell surface, NMDAR antibodies cause a reversible and selective decrease of NMDARs [25]. This leads to a higher chance of lack of structural damage to neurons, that NMDAR patients potentially respond well to early immunosuppressive treatment, depending on the length and peculiarity of antibody exposure [26]. In the previous studies, the longest median follow-up to date was 4.9 years, with improvement in all cognitive domains but persistent deficits in memory and executive function in 43 NMDAR patients [10]. In contrast, our NMDAR cohort was assessed at a median of 6.9 years after symptom onset and showed no neurocognitive impairment other than pathological AFT. This may indicate that neurocognitive recovery may be more substantial in the long-term than was previously assumed. However, a “good” functional outcome does not always guarantee complete recovery, as long-term cognitive impairments and fatigue may occur [7]. However, for patients with VGKC antibodies targeting ion channels and those with GAD antibodies targeting intracellular antigens, a less effective response to immunotherapy was described [13, 27]. These different group results between the NMDAR and non-NMDAR group, depending on the antibody type, were also seen in olfactory function/impairment, while both cognition and olfaction are related to hippocampal structures [28]. Particularly, the non-NMDAR group had significant lower odour discrimination values compared to the NMDAR group, considering that odour discrimination is related as a higher cognitive function. [28, 29]

Second, patients without limbic MRI pathologies had significant better results in visual figural functions and incidental learning compared to those with limbic MRI pathologies. Moreover, if the limbic MRI pathology was on the right hemisphere, both the visual figural function and the incidental learning capabilities were significantly lower compared to patients with normal MRI. This was observed in the complete AE group and the subgroup analyses. The significant better results in the NMDAR group may be attributable to a lesser extent of limbic damage, as discussed before. Finke and colleagues reported a correlation between verbal memory performance and total left hippocampal volume as well as presubiculum in 40 NMDAR patients. However, no significant correlations between hippocampal volumes and visuospatial memory performance were found in this and another previous study [1, 5]. Correspondingly, Wagner et al. could show a predominant affection of the amygdala in the early disease stage of VGKC- and GAD-associated AE, which resolved during the later course of disease [30]. Persisting MRI abnormalities have been observed following VGKC- and GAD-antibody positive encephalitides; however, no correlations between MRI laterality and neuropsychological outcomes were reported in an earlier study [14]. Of note, Wagner et al. found a negative correlation of diffusivity parameters with figural memory performance located mainly in the right temporal lobe in GAD patients in 2015 [2]. It is therefore a new finding of our study that abnormalities in the right hippocampus, particularly in GAD and VGKC patients, are associated with poorer performance on the RVDLT and incidental learning.

Third, we would like to discuss the RVDTL as a tool for right-hippocampal damage. Currently, there is a lack of neuropsychological tests to assess right temporal lobe memory function in patients with temporal lobe epilepsy (TLE) [23]. Typically, the Wechsler Memory Scale or the Rey Complex Figure Test is used for non-verbal memory function; however, these tests are considered inadequate for lateralising the epileptic focus in epilepsy surgery candidates with unilateral right TLE [23, 31, 32]. As discussed by Benger et al., a number of studies have failed to find differences, e.g., in face memory between left and right TLE patients and in pre- and postoperative face recognition after right temporal lobectomy. We suggest that the RVDLT may be used as a tool to detect right-hippocampal dysfunction, analogous to the AFT established by Benger et al. in 2009, as it is known that the right temporal lobe represents face recognition memory and the RVDLT correlates with white matter structure [23, 33–35]. In 14 healthy subjects, Begrè et al. were able to show a significant and direct relationship between 11 clusters of intervoxal coherence using diffusion tensor imaging in the left and right dorsal hippocampal commissure, posterior cingulate, right medial orbitofrontal regions, and other limbic brain areas with visual memory performance. [35]

In relation to the aforementioned findings, we acknowledge the various methodological limitations of this study. The most significant limitation concerns the small sample size and the heterogeneity of different antibody types, which affects the interpretation of our findings. For upcoming studies, neurocognitive sequelae after AE should be assessed prospectively in larger prospective multi-centre, case–control studies with sufficiently large subgroups defined by AE antibodies [36]. To improve understanding of neurocognitive function after AE, it is advisable to assess the neurocognitive battery not only in the long term, but also in the early treatment phase. Furthermore, sequential MRI studies should be performed between disease onset and follow-up to characterise limbic changes over time and in more detail. In our study, we did not distinguish between patients with anti-LGI1 and anti-Caspr2 among our cohort of VGKC-positive patients, although this is meanwhile established [37]. We have added one antibody-negative patient with AE to the non-NMDAR group as we focussed on characterising NMDAR and non-NMDAR subgroups. However, it is possible that autoimmune encephalitis in seronegative patients is mediated by NMDAR-like or other unknown cell surface AE antibodies, potentially resulting in effects on neurocognitive function comparable to NMDAR patients. Another limitation is the significant higher age of the non-NMDAR group compared to the NMDAR group. The higher age might affect neurocognitive function negatively [38–40]. We did not apply age correction and sensitivity analyses due to the small sample size. Moreover, as there have been no previous measurements of incidental learning levels in the Digit Symbol Substitution Test, there are no values to compare. Currently, it is recommended to validate the results of incidental learning in the DSST using healthy cohort. Finally, the selection of our comparison group of bacterial meningitis is debatable. As alternative, patients with Herpes simplex virus (HSV) encephalitis could serve as an appropriate control population [41, 42]. However, due to the known higher mortality rate compared to AE and its very low incidence, we were concerned that we would not be able to recruit enough control subjects [41–43]. In future, larger studies on cognitive functions in AE patients, patients affected by HSV encephalitis may also serve as a control group.

## Conclusion

The results of our study show mainly normal neurocognitive functions after NMDAR encephalitis in the long term. However, patients with non-NMDAR autoimmune encephalitis have impaired right-hippocampal neurocognitive functions and reduced incidental learning capacities, most likely due to structural damages of the limbic system. Additionally, we propose the RVDLT as a novel tool for detecting right-hippocampal dysfunction.

## Supplementary Information

Below is the link to the electronic supplementary material.

Supplementary file1 (DOCX 2132 KB)

## Data Availability

The data used in this article were provided by the Department of Neurology, University Hospital Dresden, Dresden, Germany. Anonymized data are available on reasonable request.

## References

[1] Finke C, Kopp UA, Pajkert A, Behrens JR, Leypoldt F, Wuerfel JT, Ploner CJ, Prüss H, Paul F (2016) Structural hippocampal damage following anti-N-Methyl-D-aspartate receptor encephalitis. Biol Psychiatry 79(9):727–734. 10.1016/j.biopsych.2015.02.024. (**Epub 2015 Feb 26 PMID: 25866294**) 25866294 10.1016/j.biopsych.2015.02.024

[2] Wagner J, Schoene-Bake JC, Witt JA, Helmstaedter C, Malter MP, Stoecker W, Probst C, Weber B, Elger CE (2016) Distinct white matter integrity in glutamic acid decarboxylase and voltage-gated potassium channel-complex antibody-associated limbic encephalitis. Epilepsia 57(3):475–483. 10.1111/epi.13297. (**Epub 2016 Jan 8 PMID: 26749370**) 26749370 10.1111/epi.13297

[3] Dalmau J, Gleichman AJ, Hughes EG, Rossi JE, Peng X, Lai M, Dessain SK, Rosenfeld MR, Balice-Gordon R, Lynch DR (2008) Anti-NMDA-receptor encephalitis: case series and analysis of the effects of antibodies. Lancet Neurol 7(12):1091–1098. 10.1016/S1474-4422(08)70224-2 18851928 10.1016/S1474-4422(08)70224-2 PMC2607118

[4] Finke C, Kopp UA, Prüss H, Dalmau J, Wandinger KP, Ploner CJ (2012) Cognitive deficits following anti-NMDA receptor encephalitis. J Neurol Neurosurg Psychiatry 83(2):195–198. 10.1136/jnnp-2011-300411 21933952 10.1136/jnnp-2011-300411 PMC3718487

[5] Finke C, Kopp UA, Scheel M, Pech LM, Soemmer C, Schlichting J, Leypoldt F, Brandt AU, Wuerfel J, Probst C, Ploner CJ, Prüss H, Paul F (2013) Functional and structural brain changes in anti-N-methyl-D-aspartate receptor encephalitis. Ann Neurol 74(2):284–296. 10.1002/ana.23932. (**Epub 2013 Jul 8 PMID: 23686722**) 23686722 10.1002/ana.23932

[6] McKeon GL, Scott JG, Spooner DM, Ryan AE, Blum S, Gillis D, Langguth D, Robinson GA (2016) Cognitive and social functioning deficits after anti-N-Methyl-D-aspartate receptor encephalitis: an exploratory case series. J Int Neuropsychol Soc 22(8):828–838. 10.1017/S1355617716000679. (**Epub 2016 Aug 22 PMID: 27546201**) 27546201 10.1017/S1355617716000679

[7] de Bruijn MAAM, Aarsen FK, van Oosterhout MP, van der Knoop MM, Catsman-Berrevoets CE, Schreurs MWJ, Bastiaansen DEM, Sillevis Smitt PAE, Neuteboom RF, Titulaer MJ, CHANCE Study Group (2018) Long-term neuropsychological outcome following pediatric anti-NMDAR encephalitis. Neurology. 10.1212/WNL.0000000000005605 29703768 10.1212/WNL.0000000000005605 PMC5980521

[8] Blum RA, Tomlinson AR, Jetté N, Kwon CS, Easton A, Yeshokumar AK (2020) Assessment of long-term psychosocial outcomes in anti-NMDA receptor encephalitis. Epilepsy Behav 108:107088. 10.1016/j.yebeh.2020.107088. (**Epub 2020 May 3 PMID: 32375094**) 32375094 10.1016/j.yebeh.2020.107088

[9] Bastiaansen AEM, van Steenhoven RW, de Bruijn MAAM, Crijnen YS, van Sonderen A, van Coevorden-Hameete MH, Nühn MM, Verbeek MM, Schreurs MWJ, Sillevis Smitt PAE, de Vries JM, Jan de Jong F, Titulaer MJ (2021) Autoimmune encephalitis resembling dementia syndromes. Neurol Neuroimmunol Neuroinflamm. 10.1212/NXI.0000000000001039 34341093 10.1212/NXI.0000000000001039 PMC8362342

[10] Heine J, Kopp UA, Klag J, Ploner CJ, Prüss H, Finke C (2021) Long-term cognitive outcome in anti-n-methyl-d-aspartate receptor encephalitis. Ann Neurol 90(6):949–961. 10.1002/ana.26241. (**Epub 2021 Oct 21 PMID: 34595771**) 34595771 10.1002/ana.26241

[11] Guasp M, Rosa-Justicia M, Muñoz-Lopetegi A, Martínez-Hernández E, Armangué T, Sugranyes G, Stein H, Borràs R, Prades L, Ariño H, Planagumà J, De-La-Serna E, Escudero D, Llufriu S, Sánchez-Valle R, Santamaria J, Compte A, Castro-Fornieles J, Dalmau J, Spanish anti-NMDAR Encephalitis Study Group (2022) Clinical characterisation of patients in the post-acute stage of anti-NMDA receptor encephalitis: a prospective cohort study and comparison with patients with schizophrenia spectrum disorders. Lancet Neurol 21(10):899–910. 10.1016/S1474-4422(22)00299-X 36115362 10.1016/S1474-4422(22)00299-X

[12] Chen X, Li JM, Liu F, Wang Q, Zhou D, Lai X (2016) Anti-N-methyl-D-aspartate receptor encephalitis: a common cause of encephalitis in the intensive care unit. Neurol Sci 37(12):1993–1998. 10.1007/s10072-016-2702-y. (**Epub 2016 Sep 12 PMID: 27620725**) 27620725 10.1007/s10072-016-2702-y

[13] Butler CR, Miller TD, Kaur MS, Baker IW, Boothroyd GD, Illman NA, Rosenthal CR, Vincent A, Buckley CJ (2014) Persistent anterograde amnesia following limbic encephalitis associated with antibodies to the voltage-gated potassium channel complex. J Neurol Neurosurg Psychiatry 85(4):387–391. 10.1136/jnnp-2013-306724. (**Epub 2014 Jan 8 PMID: 24403282**) 24403282 10.1136/jnnp-2013-306724

[14] Frisch C, Malter MP, Elger CE, Helmstaedter C (2013) Neuropsychological course of voltage-gated potassium channel and glutamic acid decarboxylase antibody related limbic encephalitis. Eur J Neurol 20(9):1297–1304. 10.1111/ene.12186. (**Epub 2013 May 17 PMID: 23678940**) 23678940 10.1111/ene.12186

[15] Graus F, Titulaer MJ, Balu R, Benseler S, Bien CG, Cellucci T et al (2016) A clinical approach to diagnosis of autoimmune encephalitis. Lancet Neurol 15:391–404. 10.1016/S1474-4422(15)00401-9 26906964 10.1016/S1474-4422(15)00401-9 PMC5066574

[16] Dale RC, Irani SR, Brilot F, Pillai S, Webster R, Gill D et al (2009) N-methyl-D-aspartate receptor antibodies in pediatric dyskinetic encephalitis lethargica. Ann Neurol 66:704–709. 10.1002/ana.21807 19938173 10.1002/ana.21807

[17] Davies G, Irani SR, Coltart C, Ingle G, Amin Y, Taylor C et al (2010) Anti-N-methyl-D-aspartate receptor antibodies: A potentially treatable cause of encephalitis in the intensive care unit. Crit Care Med 38:679–682. 10.1097/CCM.0b013e3181cb0968 20016378 10.1097/CCM.0b013e3181cb0968

[18] Dogan Onugoren M, Deuretzbacher D, Haensch CA, Hagedorn HJ, Halve S, Isenmann S et al (2015) Limbic encephalitis due to GABAB and AMPA receptor antibodies: a case series. J Neurol Neurosurg Psychiatry 86:965–972. 10.1136/jnnp-2014-308814 25300449 10.1136/jnnp-2014-308814

[19] Zigmond AS, Snaith RP (1983) The hospital anxiety and depression scale. Acta Psychiatr Scand 67(6):361–370. 10.1111/j.1600-0447.1983.tb09716.x. (**PMID: 6880820**) 6880820 10.1111/j.1600-0447.1983.tb09716.x

[20] Helmstaedter C, Lendt M, Lux S (2001) Verbaler Lern-und Merkfähigkeitstest (VLMT) *Beltz*. Göttingen, Germany

[21] Rey A (1968) Epreuves mnésiques apprentissage. In: Rey A (ed) Actualités Pedagogiques Psychologiques Memory tests and learning Recent developments in Pedagogy and Psychology. Springer, Switzerland

[22] Spreen O, Strauss E (1991) A compendium of neuropsychological tests: Administration, norms, and commentary. Oxford University Press

[23] Bengner T, Malina T (2010) Long-term face memory as a measure of right temporal lobe function in TLE: the Alsterdorfer Faces Test. Epilepsy Res 89(1):142–147. 10.1016/j.eplepsyres.2009.11.016. (**Epub 2010 Jan 19 PMID: 20034763**) 20034763 10.1016/j.eplepsyres.2009.11.016

[24] Nasreddine ZS, Phillips NA, Bédirian V, Charbonneau S, Whitehead V, Collin I, Cummings JL, Chertkow H (2005) The Montreal Cognitive Assessment, MoCA: a brief screening tool for mild cognitive impairment. J Am Geriatr Soc 53(4):695–699. 10.1111/j.1532-5415.2005.53221.x.Erratum.In:JAmGeriatrSoc. 15817019 10.1111/j.1532-5415.2005.53221.x

[25] Hughes EG, Peng X, Gleichman AJ, Lai M, Zhou L, Tsou R, Parsons TD, Lynch DR, Dalmau J, Balice-Gordon RJ (2010) Cellular and synaptic mechanisms of anti-NMDA receptor encephalitis. J Neurosci 30(17):5866–5875. 10.1523/JNEUROSCI.0167-10.2010. 20427647 10.1523/JNEUROSCI.0167-10.2010 PMC2868315

[26] Leypoldt F (2013) Autoimmune Enzephalitiden. Fortschritte der Neurologie – Psychiatrie. 10.1055/s-0033-1350145 23986460 10.1055/s-0033-1350145

[27] Malter MP, Helmstaedter C, Urbach H, Vincent A, Bien CG (2010) Antibodies to glutamic acid decarboxylase define a form of limbic encephalitis. Ann Neurol 67(4):470–478. 10.1002/ana.21917. (**PMID: 20437582**) 20437582 10.1002/ana.21917

[28] Hänsel M, Schmitz-Peiffer H, Hähner A, Reichmann H, Schneider H (2023) Olfactory dysfunction after autoimmune encephalitis depending on the antibody type and limbic MRI pathologies. Front Neurol 25(14):1225975. 10.3389/fneur.2023.1225975 10.3389/fneur.2023.1225975 PMC10486887 37693764

[29] Levy DA, Hopkins RO, Squire LR (2004) Impaired odour recognition memory in patients with hippocampal lesions. Learn Mem 11(6):794–796. 10.1101/lm.82504 15537736 10.1101/lm.82504 PMC534708

[30] Wagner J, Weber B, Elger CE (2015) Early and chronic gray matter volume changes in limbic encephalitis revealed by voxel-based morphometry. Epilepsia 56(5):754–761. 10.1111/epi.12968. (**Epub 2015 Mar 23 PMID: 25809952**) 25809952 10.1111/epi.12968

[31] Barr WB (1997) Examining the right temporal lobe’s role in nonverbal memory. Brain Cogn 35(1):26–41. 10.1006/brcg.1997.0925. (**PMID: 9339300**) 9339300 10.1006/brcg.1997.0925

[32] Lee TM, Yip JT, Jones-Gotman M (2002) Memory deficits after resection from left or right anterior temporal lobe in humans: a meta-analytic review. Epilepsia 43(3):283–291. 10.1046/j.1528-1157.2002.09901.x. (**PMID: 11906514**) 11906514 10.1046/j.1528-1157.2002.09901.x

[33] Kelley WM, Miezin FM, McDermott KB, Buckner RL, Raichle ME, Cohen NJ, Ollinger JM, Akbudak E, Conturo TE, Snyder AZ, Petersen SE (1998) Hemispheric specialization in human dorsal frontal cortex and medial temporal lobe for verbal and nonverbal memory encoding. Neuron 20(5):927–936. 10.1016/s0896-6273(00)80474-2. (**PMID: 9620697**) 9620697 10.1016/s0896-6273(00)80474-2

[34] Coleshill SG, Binnie CD, Morris RG, Alarcón G, van Emde BW, Velis DN, Simmons A, Polkey CE, van Veelen CW, van Rijen PC (2004) Material-specific recognition memory deficits elicited by unilateral hippocampal electrical stimulation. J Neurosci 24(7):1612–1616. 10.1523/JNEUROSCI.4352-03.2004. 14973245 10.1523/JNEUROSCI.4352-03.2004 PMC6730466

[35] Begré S, Kiefer C, von Känel R, Frommer A, Federspiel A (2009) Rey visual design learning test performance correlates with white matter structure. Acta Neuropsychiatr 21(2):67–74. 10.1111/j.1601-5215.2009.00361.x. (**PMID: 25384565**) 25384565 10.1111/j.1601-5215.2009.00361.x

[36] Granerod J, Ambrose HE, Davies NW, Clewley JP, Walsh AL, Morgan D, Cunningham R, Zuckerman M, Mutton KJ, Solomon T, Ward KN, Lunn MP, Irani SR, Vincent A, Brown DW, Crowcroft NS, UK Health Protection Agency (HPA) Aetiology of Encephalitis Study Group (2010) Causes of encephalitis and differences in their clinical presentations in England: a multicentre, population-based prospective study. Lancet Infect Dis 10(12):835–844. 10.1016/S1473-3099(10)70222-X 20952256 10.1016/S1473-3099(10)70222-X

[37] van Sonderen A, Schreurs MW, Wirtz PW, Sillevis Smitt PA, Titulaer MJ (2016) From VGKC to LGI1 and Caspr2 encephalitis: The evolution of a disease entity over time. Autoimmun Rev 15(10):970–974. 10.1016/j.autrev.2016.07.018. (**Epub 2016 Jul 30 PMID: 27485013**) 27485013 10.1016/j.autrev.2016.07.018

[38] Cohen G (1987) Speech comprehension in the elderly: the effects of cognitive changes. Br J Audiol 21(3):221–226. 10.3109/03005368709076408. (**PMID: 3304489**) 3304489 10.3109/03005368709076408

[39] See SK, Ryan EB (1996) Cognitive mediation of discourse processing in later life. J Speech Lang Pathol Audiol 20:109–109

[40] Wingfield A, Tun PA (2001) Spoken language comprehension in older adults: interactions between sensory and cognitive change in normal aging. Semin Hear. 10.1055/s-2001-15632

[41] McGrath N, Anderson NE, Croxson MC, Powell KF (1997) Herpes simplex encephalitis treated with acyclovir: diagnosis and long term outcome. J Neurol Neurosurg Psychiatry 63(3):321–326. 10.1136/jnnp.63.3.321 9328248 10.1136/jnnp.63.3.321 PMC2169720

[42] Westman G, Aurelius E, Ahlm C, Blennow K, Eriksson K, Lind L, Schliamser S, Sund F, Zetterberg H, Studahl M (2021) Cerebrospinal fluid biomarkers of brain injury, inflammation and synaptic autoimmunity predict long-term neurocognitive outcome in herpes simplex encephalitis. Clin Microbiol Infect 27(8):1131–1136. 10.1016/j.cmi.2020.09.031. (**Epub 2020 Sep 23 PMID: 32979577**) 32979577 10.1016/j.cmi.2020.09.031

[43] Pietrini V, Nertempi P, Vaglia A, Revello MG, Pinna V, Ferro-Milone F (1988) Recovery from herpes simplex encephalitis: selective impairment of specific semantic categories with neuroradiological correlation. J Neurol Neurosurg Psychiatry 51(10):1284–1293. 10.1136/jnnp.51.10.1284 3225585 10.1136/jnnp.51.10.1284 PMC1032917

