# Supplementary material for: Hippocampal dysfunction after autoimmune encephalitis depending on the antibody type
Source: J Neurol. 2025 Feb 1;272(2):175. doi: 10.1007/s00415-024-12742-1 (PMC11787161; doi:10.1007/s00415-024-12742-1)
Supplement: Supplementary file 1 — Supplementary file1 (DOCX 2132 KB) [file 415_2024_12742_MOESM1_ESM.docx]

Supplementary Material

Hippocampal dysfunction after autoimmune encephalitis depending on the antibody type

Martin Hänsel^1,2^ , Heinz Reichmann^1^, Antje Hähner^3^, Henning Schmitz-Peiffer^1,†^, Hauke Schneider ^1,4,†,*^

^1^Department of Neurology, University of Dresden, Dresden, Germany

^2^Department of Internal Medicine, GZO - Zurich Regional Health Center, Wetzikon, Switzerland

^3^Smell and Taste Clinic, Department of Otorhinolaryngology, Medical Faculty Carl-Gustav Carus, Technical University of Dresden, Dresden, Germany

^4^Department of Neurology, Augsburg University Hospital, Augsburg, Germany

^†^Authors contributed equally

*** Correspondence:** Corresponding Author: hauke.schneider@uk-augsburg.de

# Methods

# Inclusion criteria

Patients had to be 18 years of age or older, not pregnant, and consent to participate in the study. Other inclusion criteria for the control group of PC patients were treatment in an intensive care unit, surgical treatment of an infectious focus (sinugen, otogen), age at diagnosis 40 ±15 years, and detection of Streptococcus pneumoniae in blood cultures. The flowchart of the enrolment process is shown in Supplementary Figure S1.

**Detection of antibodies**

Antibodies to NR1/NR2B heteromers of the NMDAR were found through indirect immunofluorescence on NR1/NR2B transfected human embryonic kidney cells for AE patients diagnosed in 2010 and 2011.^1-2^ For all other AE patients, the antibodies were detected through indirect immunofluorescence on commercially available mouse brain tissue and cell-based assays (Euroimmun, Lübeck, Germany).^3^ The tests involved modified human embryonic kidney cells (HEK 293) with plasmids holding neuronal substances such as N-methyl-D-aspartate receptor (NMDAR), which consists of NR1 subunits alone, and NMDAR (NR1-NR2), glutamic acid decarboxylase (GAD) GAD65, GAD67, leucine-rich glioma inactivated 1 protein (LGI1), contactin-associated protein-like 2 (CASPR2), AMPAR1, AMPAR2, GABABR, glycine receptor (GlyR).

# Neurocognitive battery

We used the following sequence of neurocognitive tests for each patient to provide a standardized assessment: 1. anamnesis relating to neurocognitive deficits, 2. Aphasia score, 3. Hospital anxiety and depression scale (HADS), 4. Verbal Learning Memory Test (VLMT), 5. Rey Visual Design Learning Test (RVDLT), 6. Alsterdorfer Faces Test (AFT), 7. Digit Symbol Substitution Test (DSST) with recall, 8. VLMT-long delay, 9. RVDLT-long delay, 10. Montreal Cognitive Assessment (MoCA)

**Aphasia score:** To test for aphasia and exclude aphasic patients, all patients had to score at least 11 points (Supplementary Figure S2).

**HADS-A/D:** The test screened for depression and anxiety.^4^ A questionnaire with alternating questions (each subscale with 7 items) on depression (HADS-D) and anxiety (HADS-A) in the last week had to be completed. For each item, a score between 0 (yes, definitely) and 3 (no, not at all) was possible, with a maximum of 21 points. A higher score indicates greater distress. A score of 0 to 7 is considered normal, a score of 8 to 10 is suspicious, and a score of >10 indicates suspicion of depression (HADS-D) or anxiety (HADS-A) with a specificity of 87/85% and a sensitivity of 72/80%.^5^

**Verbal Learning Memory Test:** The test consists of 2 word lists (A and B).^6^ Each list contains 15 words. The examiner read the items from list A 5 times at 2-second intervals, and the patients had to identify the maximum number of words after each reading. In the 6th round, list B was read to distract the patients. The patients also had to notice as many words as possible. In round 7 the patients had to memorise all the words from the A list without being told. After 20 to 30 minutes, the patients were asked to recall list A (long delay). They also needed to recognise all 15 items from list A using a sample of 50 words. Overall performance, recognition performance and long delay were measured.

**Rey Visual Design Learning Test:** In an interval of 2 seconds, 15 different figures were shown in 5 rounds.^7,8^ After each of the 5 rounds, the patient had to draw as many figures as possible. The patient had to remember and draw all the figures with a delay of 20-30 minutes. In addition, they had to recognise the 15 figures out of a total of 30 different figures. Measurements were made of overall performance, recognition performance and long delay.

**Alsterdorfer Faces Test:** The Alsterdorfer Face Test is a face recognition test consisting of 2 steps.^9^ Step 1 is a learning phase in which different black-and-white portraits were shown every 5 seconds. After 20 faces, step 2 began. During this control phase, 40 faces were shown, 20 familiar (from step 1) and 20 unfamiliar. The patients had to decide which 20 faces were new and which 20 had been shown in step 1. The parameter "corrected number" [%] was measured and is normal at >75%.

**Digit Symbol Substitution Test:** In the DSST, the numbers 1-9 are assigned different symbols. After a first learning phase, patients had to match as many symbols as possible to the given numbers in 90 seconds. In the second phase, patients had to recapitulate the correct 9 symbols for the 9 numbers to check incidental learning.

**Montreal Cognitive Assessment:** The MoCA is a screening tool for mild cognitive impairment (MCI).^10^ Several cognitive domains are assessed: visuoconstructive ability, memory, language, attention and concentration, executive function, conceptual ability, numeracy and orientation. The maximum score is 30 points. A score of ≥26 points is normal. A score of ≤25points indicates for MCI or early dementia with a sensitivity/specifity of 90/87%.

# Results

**CSF and serum results at diagnosis and post-acute phase (<6 months)**

The CSF and serum results at diagnosis and post-acute phase (<6 months) are presented in the Supplementary Table 1.

**Further investigations at follow-up in AE and PC patients**

All patients had at least 11 points at the aphasia score. Median HADS-D scores were not significantly different (p = 0.229) between groups (AE 3 points, range 1-12; PC 5 points, range 1-17). One GAD patient in the AE group and 2 in the PC group had elevated HADS-D scores (>10 points). Median HADS-A score was 5 points (range 1-13) in AE group and 5.5 points (range 2-12) in PC group (p = 0.774). 10 AE and 10 PC patients had no significantly elevated HADS-A scores.

**Further investigations at follow-up in NMDAR and non-NMDAR patients**

The aphasia score was at least 11 points in all patients. Only one GAD patient had an elevated HADS-D score (12 points), all other patients in the non-NMDAR group (median 3 points, range 1-12) and the NMDAR group (median 2.5 points, range 1-4) had non-significantly (p = 0.471) different normal results. The Median HADS-A score was 5.5 points (range 2-10) in NMDAR group and 5 points (range 1-13) in non-NMDAR group (p = 0.989). One NMDAR patient (10 points) and one non-NMDAR patient with antibodies against GAD (9 points) had a suspicious result, one non-NMDAR patient (GAD antibodies) had an elevated HADS-A level (13 points).

**The distribution of neurocognitive results**

VLMT overall, VLMT recognition, RVDLT overall, RVDLT recognition, AFT and DSST were proximately normally distributed in both the AE and PC groups, as assessed by the Shapiro-Wilk test, p > 0.05. DSST incidental was approximately normally distributed for the AE group (p = 0.157) but not for the PC group (p = 0.033). The MoCA was approximately normally distributed for the PC group (p = 0.176) but not for the AE group (p = 0.003).

For the NMDAR and non-NMDAR groups, VLMT overall, VLMT recognition, VLMT long delay, RVDLT overall, RVDLT long delay, AFT, DSST, DSST incidental and MoCA were normally distributed as assessed by the Shapiro-Wilk test. RVDLT recognition was normally distributed for the non-NMDAR group (p = 0.615), but not for the NMDAR group (p = 0.024).

**References:**

1. Dale RC, Irani SR, Brilot F, Pillai S, Webster R, Gill D, et al. N-methyl-D-aspartate receptor antibodies in pediatric dyskinetic encephalitis lethargica. *Ann Neurol*. (2009) 66:704-9. doi: 10.1002/ana.21807
2. Davies G, Irani SR, Coltart C, Ingle G, Amin Y, Taylor C, et al. Anti-N-methyl-D-aspartate receptor antibodies: A potentially treatable cause of encephalitis in the intensive care unit. *Critical Care Medicine*. (2010) 38:679-82. doi: 10.1097/CCM.0b013e3181cb0968
3. Dogan Onugoren M, Deuretzbacher D, Haensch CA, Hagedorn HJ, Halve S, Isenmann S, et al. Limbic encephalitis due to GABAB and AMPA receptor antibodies: a case series. *J Neurol Neurosurg Psychiatry*. (2015) 86:965-72. doi: 10.1136/jnnp-2014-308814
4. Zigmond AS, Snaith RP. The hospital anxiety and depression scale. *Acta Psychiatr Scand*. 1983 Jun;67(6):361-70. doi: 10.1111/j.1600-0447.1983.tb09716.x.
5. Herrero MJ, Blanch J, Peri JM, De Pablo J, Pintor L, Bulbena A. 2003. A validation study of the hospital anxiety and depression scale (HADS) in a Spanish population. *General Hospital Psychiatry*, 25(4):277–283 DOI: 10.1016/S0163-8343(03)00043-4.
6. Helmstaedter, C., Lendt, M. & Lux, S. Verbaler Lern-und Merkfähigkeitstest (VLMT) Beltz. Göttingen, Germany (2001)
7. Rey, A. (1968). Epreuves mnésiques et d'apprentissage. Actualités Pedagogiques et Psychologiques [Memory tests and learning. Recent developments in Pedagogy and Psychology]. Neuchatel, Switzerland : Delachaux & Neistle
8. Spreen, O., & Strauss, E. (1991). A compendium of neuropsychological tests: Administration, norms, and commentary. Oxford University Press
9. Bengner T, Malina T. Long-term face memory as a measure of right temporal lobe function in TLE: the Alsterdorfer Faces Test. *Epilepsy Res*. 2010 Mar;89(1):142-7. doi: 10.1016/j.eplepsyres.2009.11.016.
10. Nasreddine ZS, Phillips NA, Bédirian V, Charbonneau S, Whitehead V, Collin I, Cummings JL, Chertkow H. The Montreal Cognitive Assessment, MoCA: a brief screening tool for mild cognitive impairment. *J Am Geriatr Soc*. 2005 Apr;53(4):695-9. doi: 10.1111/j.1532-5415.2005.53221.x.

# Figures

**Supplementary Figure S1**


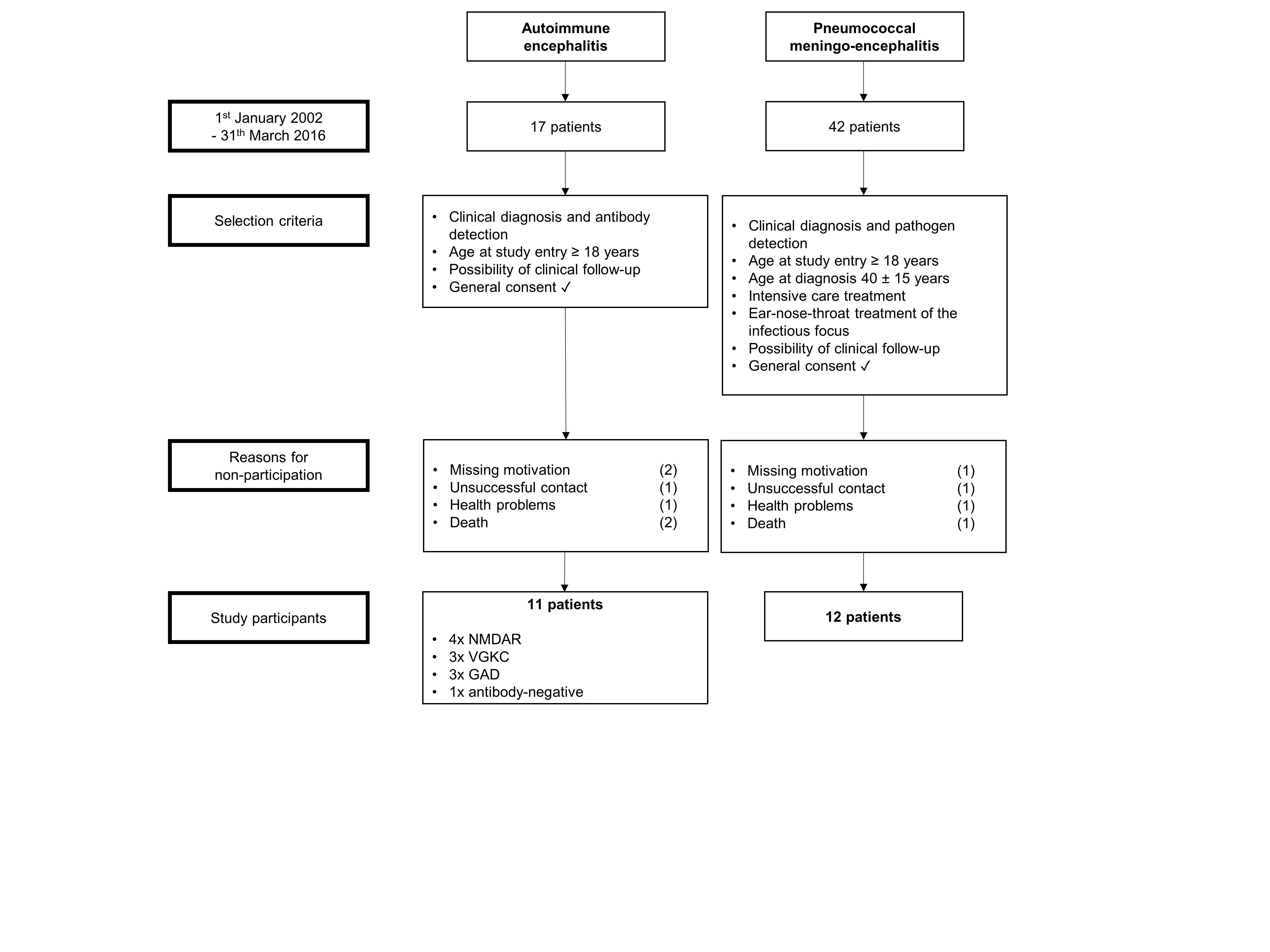


**Supplementary Figure S2: Aphasia score**
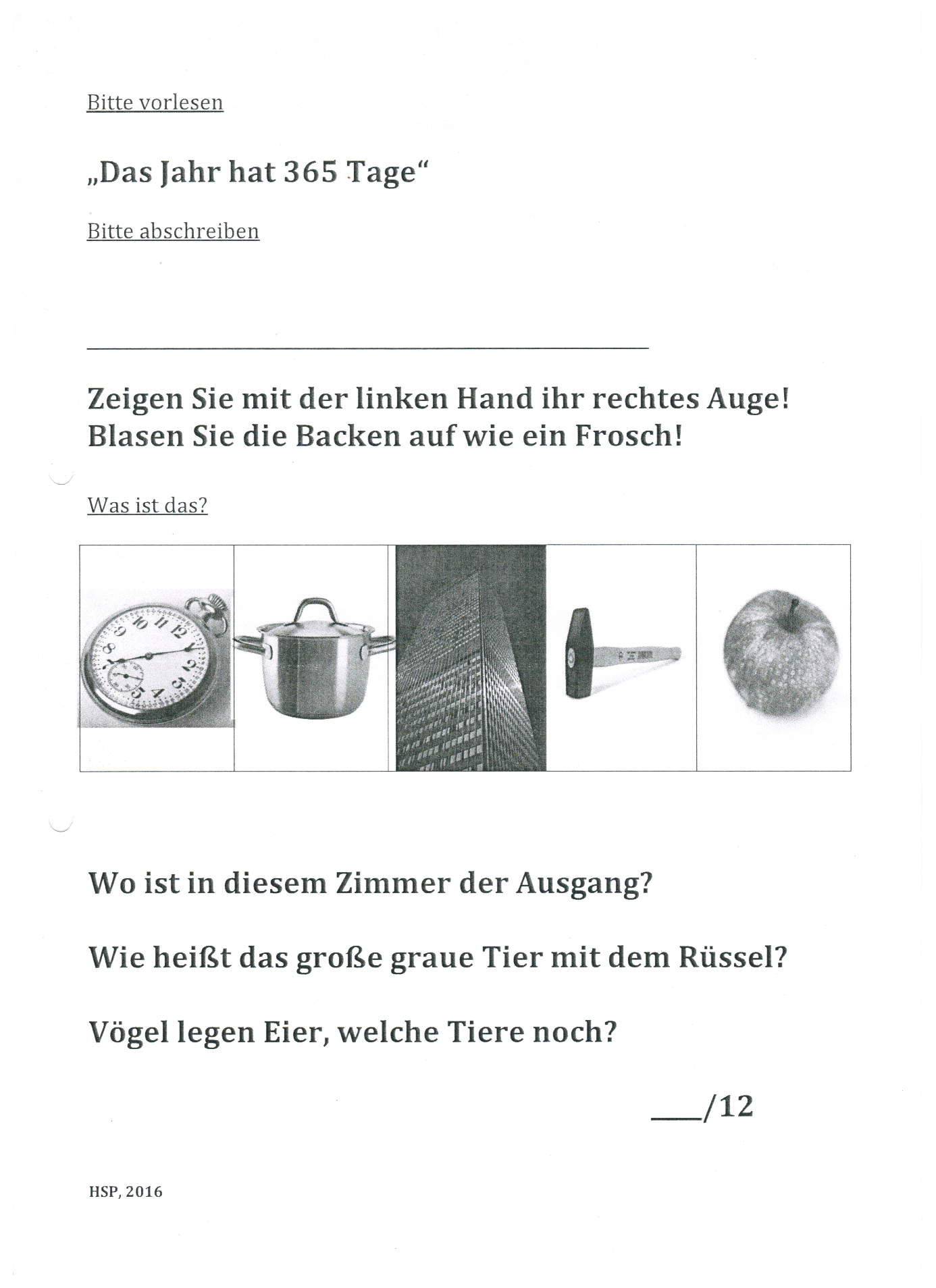


**Supplementary Table 1: CSF and serum results at diagnosis and post-acute phase (<6 months) in NMDAR and non-NMDAR groups**

| Patient | 1 | 2 | 3 | 4 | 5 | 6 | 7 | 8 | 9 | 10 | 11 |
| --- | --- | --- | --- | --- | --- | --- | --- | --- | --- | --- | --- |
| Antibody | NMDAR | NMDAR | NMDAR | negativ | VGKC | GAD | GAD | VGKC | VGKC | GAD | NMDAR |
| Acute phase (Serum/CSF) | -/+ | +/+ | +/NL | -/- | +/- | +/+ | +/+ | +/- | +/+ | +/+ | +/+ |
| Post-acute phase (Serum/CSF), (<6 months) | -/+ | -/+ | -/NL | -/NL | -/- | +/+ | -/NL | -/NL | +/NL | +/NL | -/NL |
| *+, Positive detection; -, Negative (no) detection; CSF, Cerebrospinal fluid; GAD,* *glutamate acid decarboxylase; NL, No lumbar puncture; NMDAR, N-methyl-D-aspartate Receptor; non-NMDAR, without N-methyl-D-aspartate Receptor; VGKC, voltage-gated potassium channels* | | | | | | | | | | | |
